# Adolescent Mental Health Problems and Adult Human Capital: Findings From the South African Birth to Twenty Plus Cohort at 28 Years of Age

**DOI:** 10.1016/j.jadohealth.2021.04.017

**Published:** 2021-11

**Authors:** Linda M. Richter, Marilyn N. Ahun, Sahba Besharati, Sara N. Naicker, Massimiliano Orri

**Affiliations:** a DSI-NRF Centre of Excellence in Human Development, University of the Witwatersrand, Johannesburg, South Africa; b Department of Social and Preventive Medicine, Université de Montréal School of Public Health, Montréal, Canada; c Department of Psychology, School of Human and Community Development, University of the Witwatersrand, Johannesburg, South Africa; d McGill Group For Suicide Studies, Douglas Mental Health University Institute, Department of Psychiatry, McGill University, Montreal, Canada

**Keywords:** Adolescent, Internalizing problems, Externalizing problems, Human capital, Birth cohort, South Africa

## Abstract

**Purpose:**

We investigated associations between adolescent internalizing and externalizing problems and adult human capital in a non-Western setting. Little is known about adolescent mental health problems and adult outcomes in low- and middle-income countries, many of which are characterized by high levels of adversities.

**Methods:**

Data came from the Birth to Twenty Plus cohort, started in Soweto, Johannesburg, South Africa, in 1990. We estimated associations of internalizing and externalizing problems at the age of 14 years with self-reported educational, employment, welfare receipt, psychosocial (psychological distress, criminality, substance use), interpersonal (social isolation, intimate partner violence, partnership status), and HIV outcomes at the age of 28 years.

**Results:**

Adolescents with high internalizing problems were less likely to have completed secondary school or be formally employed and more likely to report psychological distress. Those with high levels of externalizing problems were more likely to report adulthood criminal activity and substance use. We found significant associations between internalizing and externalizing problems and intimate partner violence. There was no association between adolescent mental health problems and welfare receipt, HIV, social isolation, or partnership status. Men were more likely to report incomplete secondary education, no formal employment, criminality and substance use, social isolation, and no serious relationship, whereas women were more likely to experience psychological distress and be in receipt of welfare.

**Conclusions:**

Adolescent mental health problems are associated with long-term negative adult functioning under varying socioeconomic conditions. Interventions to recognize and address youth mental health problems in low- and middle-income countries are needed to avert serious adverse adult and societal consequences.

**Implications and Contribution**

Adolescent mental health problems are associated with negative outcomes across most spheres of adult life – education, employment, psychosocial functioning, and interpersonal relationships –with differences between men and women. Contextually relevant interventions are needed to significantly reduce the prevalence of adolescent mental health problems and protect human capital development in low- and middle-income countries.

Mental health problems are a leading cause of disability among children and adolescents with overall worldwide prevalence at 10%–20% [1]. Estimates for internalizing symptoms (i.e., emotional symptoms such as anxiety or depression which reflect internal distress) [2] range from 1.3% to 6.5% and for externalizing symptoms (i.e., behaviors such as conduct disorder and hyperactivity/impulsivity which are overt and can result in conflict with others) from 2.1% to 5.7% [3]. Low- and middle-income countries (LMICs) are home to almost 90% of children younger than the age of 18 years; youth mental health problems account for nearly a third (29%) of years lost due to disability [4]. In Africa, mental health problems are the leading cause of years lost due to disability and account for 208 disability-adjusted life years among 10- to 24-year-old individuals [4]. Prevalence estimates show that 13.4% of children and adolescents (5–16 years) worldwide and 14.3% of children and adolescents in sub-Saharan Africa experience some level of mental health problems [[3], [4], [5]]. Despite these numbers, few studies have examined longer-term personal and societal consequences of adolescent mental health problems in LMICs, resulting in the neglect of adolescent mental health and nominal efforts to recognize, treat, and ameliorate adolescent distress and prevent adverse outcomes in later life.

There is robust evidence from high-income countries (HICs) that adolescents who experience mental health problems are more likely to have poorer human capital outcomes (i.e., economic [e.g., higher educational attainment and employment], psychosocial [e.g., psychological distress, substance abuse and criminality], and interpersonal [e.g., healthy social relationships]) than those who do not [[6], [7], [8]]. For example, depression severity at the ages of 14–16 years was associated with higher rates of mental health problems, substance abuse/dependence, lower educational attainment, poorer economic circumstances, and more adverse partnership outcomes (e.g., sole parenthood, intimate partner violence [IPV]) at the ages of 30 and 35 years in a New Zealand birth cohort [7]. These poor outcomes diminish the personal and social resources constituting human capital. It is particularly important to address this issue in LMICs, where human capital development is critical to national socioeconomic progress and to individual fulfillment. It is also important theoretically to distinguish the impact of adolescent mental health problems on human capital from the impact of high levels of adversity and socioeconomic disadvantage [1].

Only a handful of studies have explored these associations in LMIC contexts. Data, mostly from cross-sectional and retrospective studies and some in the South African context, show that adolescent mental health problems are significantly associated with HIV status, substance misuse, delinquency, interpersonal violence, and lower levels of educational attainment and social support [[9], [10], [11], [12], [13]]. We found no studies that prospectively investigated human capital outcomes of adolescent mental health problems in sub-Saharan Africa. The lack of robust longitudinal data in these studies makes it difficult to ascertain the direction of associations between mental health problems and human capital outcomes. The only prospective LMIC-based study used data from the Brazilian 1993 Pelotas birth cohort and found that children with behavioral problems at 11 years of age were more likely to engage in criminal behavior, experience emotional disorders, and not be in education, employment, or training at the ages of 22–24 years [14].

Estimating the personal and societal impact of youth mental health problems is important to inform policy and to motivate the development of interventions appropriate to specific country contexts. To this aim, we used longitudinal data from the South African Birth to Twenty Plus study (Bt20+) – a birth cohort of individuals who grew up and live amidst widespread sociopolitical instability, economic stress, violence, and other life adversities – to estimate the impact of adolescent internalizing and externalizing problems on a range of adult human capital outcomes. Based on longitudinal findings from HICs and cross-sectional findings from LMICs, we hypothesized that adolescents experiencing internalizing symptoms would be more likely to experience higher levels of psychological distress and social isolation in young adulthood. We also hypothesized that externalizing symptoms would be associated with higher levels of substance use and criminality. Finally, we expected both internalizing and externalizing symptoms to be associated with lower levels of educational attainment and employment. As previous studies reported sex differences in the prevalence of internalizing (higher in female than male individuals) and externalizing (higher in male than female individuals) symptoms [15], and in human capital outcomes (e.g., substance use more prevalent in male than female individuals) [16], we explored interactions with sex and systematically reported stratified analyses by sex.

## Methods

### Participants

The Bt20+ study enrolled all singleton children born during a 7-week period in 1990, with the aim of describing the effects of rapid urbanization on child development consequent to sociopolitical change [17]. Pregnant women (N = 3,273) were recruited, and families and children have been followed up more than 22 times to the age of 28 years. Ethical approval was obtained from the Human Research Ethics Committee of the University of the Witwatersrand, Johannesburg (Ref. No. M010556). A variety of data have been collected, including social and economic circumstances, family relationships, growth, health, adjustment, schooling, and employment. Consensual agreement on phrasing of questions was reached where different interview languages (e.g., Zulu, Sotho, English) were required. Data collection techniques included parent-report, self-report, and interviewer-administered assessments. From 15 years of age, data were collected through audio-assisted computer methods, which allow respondents to listen to prerecorded survey questions through headphones while responding using a touch screen or keypad. This analysis was based on N = 1,391 participants (42% of the initial sample; 730%, 52.5%, female individuals) with available data at the age of 28 years for at least one of the study outcomes (Table 1). Included and nonincluded participants differed on a range of sociodemographic variables, as shown in Table 1.

**Table 1:** Sociodemographic characteristics of the study sample

	Included N = 1,391	Nonincluded N = 1,882	*p* value
Female sex, n (%)	730 (52.5)	949 (50.4)	.260
Birth order, n (%)			
First born	520 (37.4)	678 (36.0)	.689
Second born	408 (29.3)	580 (30.8)	
Third born	241 (17.3)	338 (18.0)	
Fourth or later	222 (16.0)	286 (15.2)	
Birth weight in kilograms, mean (SD)	3.0 (.50)	3.1 (.52)	.049
Intelligence at the age of 7 years, mean (SD)	-.10 (.92)	.17 (1.10)	<.001
Adverse childhood experiences quartiles, n (%)			<.001
1st quartile	148 (10.7)	719 (48.5)	
2nd quartile	402 (29.2)	341 (23.0)	
3rd quartile	507 (36.8)	278 (18.7)	
4th quartile	320 (23.2)	146 (9.8)	
Maternal age at birth of the index child, mean (SD)	25.77 (6.28)	26.11 (5.93)	.109
Maternal years of schooling at childbirth, mean (SD)	9.61 (2.55)	9.51 (3.32)	.379
Paternal years of schooling at childbirth, mean (SD)	10.52 (2.52)	10.65 (3.03)	.250
Crowding, people per room, mean (SD)	3.55 (1.76)	3.00 (1.48)	.001
Maternal depression score at age 6 months, mean (SD)	14.66 (7.48)	14.36 (7.76)	.041
Assets quintiles at the age of 12 years, n (%)			
1st quintile	242 (23.7)	105 (20.3)	<.001
2nd quintile	177 (17.3)	121 (23.4)	
3rd quintile	296 (29.0)	63 (12.2)	
4th quintile	112 (11.0)	109 (21.0)	
5th quintile	195 (19.1)	120 (23.2)	

SD = standard deviation.

The table reports the main sociodemographic characteristics of the study sample. For adverse childhood experience and assets, we computed the sum of adverse experiences (or of the assets) and divided the distribution in quintiles.

### Assessment of adolescent mental health problems

Mental health problems in adolescence were assessed using the Youth Self-Report [18], a validated scale completed by adolescents at the age of 14 years. Internalizing problems were captured by 24 items (alpha = .86) such as “I am unhappy” and “I worry a lot” and externalizing problems by 31 items (alpha = .84) such as “I break rules at home, school, or elsewhere” and “I physically attack other people.” Note that the original scales include more items than those available in the Bt20+ cohort. Items were answered referring to the previous 6 months and rated on a 3-point scale (not true, somewhat/sometimes true, very/often true). Scores on the two variables were standardized. The correlation between internalizing and externalizing problems was r = .46.

### Assessment of adult human capital outcomes

The following outcomes were self-reported by participants at the age of 28 years referring to the previous 12 months (unless otherwise specified):

#### Educational and employment

Participants reported their highest school grade attained, dichotomized into complete (coded 0) or incomplete [1] secondary education. Participations reported whether they were formally employed (i.e., had a work contract) (coded 0) versus not formally employed (coded 1). The welfare receipt in the form of a Child Support Grant (yes, coded 1, vs. no, coded 0) was recorded from data consented to by participants, supplied by the South African Social Security Agency and linked to the cohort through identity numbers.

#### Psychosocial

Psychological distress was assessed using the WHO's Self-Reporting Questionnaire [19] that includes 20 binary items assessing symptoms experienced during the month, such as “Do you feel nervous, tense or worried?” and “Do you sleep badly?” summed to obtain a total psychological distress score (alpha = .93). Participants in the top 20% of symptoms score were considered as having high psychological distress. Substance abuse was derived from a combination of reporting alcohol use more than 2 to 3 times a week, and the current use of nonmedical drugs (including marijuana) scored as yes (1) or no (0). Criminality was assessed by asking participants whether, in the last year, they had been arrested, detained, jailed, or committed a crime without being caught, for example, stolen a car/motorbike, stolen in a shop or from a person, sold drugs or stolen goods, set property on fire or damaged/destroyed property, assaulted someone, or forced someone to have sex. A positive answer to any of the questions was coded 1 versus 0.

#### Interpersonal

Social isolation was assessed using eight items based on the Inventory of Socially Supportive Behaviors [20], such as “how often you had someone who would listen to you when you needed to talk” and “had someone you trust to talk with about your problems.” Items were answered on a 5-point scale from *never* to *always* and summed (alpha = 0.91). We dichotomized scores to identify participants who reported high levels of social isolation (scoring at the bottom decile of the distribution, coded 1) versus those reporting lower levels (coded 0). To determine relationship status, we asked participants “Have you been in a serious relationship in the past 12 months?” (yes, coded 0, vs. no, 1). IPV was assessed using the 15 questions about physical violence from the Conflict Tactics Scale, short-version (alpha = .82) [21], such as “My partner punched or hit me with something that could hurt,” “My partner had a broken bone from a fight with me,” and “I used threats to make my partner have sex.” Questions were answered on a 7-point scale assessing the frequency of each behavior. We included an affirmative report at least once in the past year. We distinguished between four mutually exclusive patterns of IPV: victimization (i.e., participant only reported being the subject of violence), abuse (i.e., participant only reported perpetrating violence toward their partner), both abuse and victimization, and no report of IPV.

#### HIV status

HIV status was assessed by asking “Have you ever tested positive for HIV?” coded 1 if yes and 0 if no.

### Covariates

Covariates were included in the analyses based on existing literature with some evidence of association with adverse outcomes. Sex; birth order; birth weight; intelligence measured using the Raven's Colored Progressive Matrices, a nonverbal cognitive test with less cultural bias in multilingual settings such as South Africa compared with language-based assessments [22], administered by a trained researcher at participant age seven; maternal age at childbirth; maternal and paternal years of schooling; household crowding (i.e., average number of people per room living in the participant's house in early childhood); maternal depression at the child’s age of 6 months, measured using the Pitt Inventory [23]; socioeconomic status at the child’s age of 12 years, measured as wealth quintiles derived from a list of assets (e.g., TV, fridge, car, phone), as per the methodology of Filmer and Pritchett [24]; and adverse childhood experiences, assessed using prospective measures of several adverse experiences (including sexual and physical abuse, parental death, child separation from the parents, chronic unemployment of the members of the household, exposure to violence, and substance abuse in the household), were reported by caregivers at the ages of 5, 7, and 11 years and by participants at the ages of 11 and 13 years [25]. The sum of any reported adverse childhood experience between the ages of 5 and 13 years was computed and split into quintiles.

### Statistical analysis

We estimated associations between internalizing and externalizing problems and each adult human capital outcome using logistic regression, with each outcome analyzed in a separate regression. Analyses were performed for the whole sample (sex combined), as we found no evidence of significant sex interaction. However, for descriptive purposes, we also report sex-specific analyses. Sex-adjusted and fully adjusted models (i.e., adjusted for the selected covariates and in which internalizing and externalizing problems were considered in the same model) were estimated. Therefore, odds ratios (ORs) for internalizing problems from the fully adjusted models indicate the contribution of internalizing problems independently from externalizing problems and vice versa. The proportion of missing data among outcomes ranged from .1% (employment) to 9.7% (HIV); IPV had 29% missing data, as participants who were not currently in a relationship did not answer the questions; welfare receipt had 27% missing data because not all participants (e.g., those without children) were eligible (Appendix Table 1). To ensure consistency, we used multiple imputation to deal with missing data (except for IPV and welfare), so that our models were estimated across 50 imputed data sets and the estimates pooled. In the main analysis, we estimated associations for all individuals (N = 1,391) having outcome data. Of them, 896 also have data on adolescent mental health problems; data for the remaining participants were supplemented with multiple imputation. However, we also estimated all associations in the subsample with data for both adolescent internalizing and externalizing problems and outcomes (N = 896) in complementary analyses.

## Results

**Figure 1:**
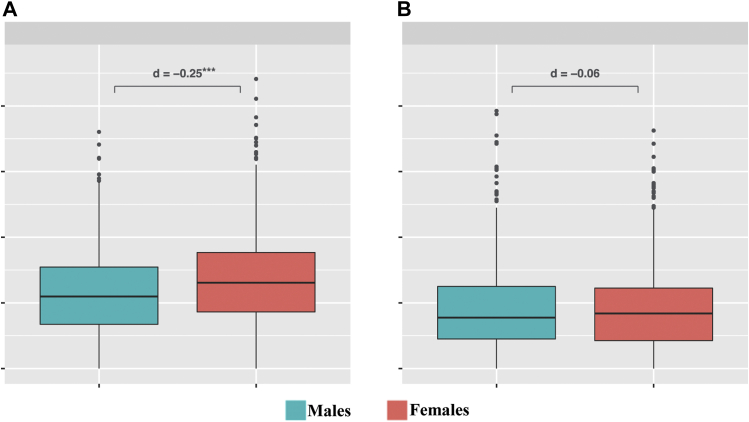
Distribution of internalizing and externalizing scores among men and women. The figure shows the distribution of internalizing (A) and externalizing (B) problem scores in men (blue) and women (red). Differences are expressed as effect size (Cohens' D), and significance was tested using t-tests. ∗∗∗*p* < .001.

The mean adolescent internalizing and externalizing problem scores were 12.8 (standard deviation = 7.0, range 0–44.1) and 9.0 (standard deviation = 6.8, range 0–39.3), respectively (Figure 1). Female adolescents reported higher numbers of internalizing problems than male adolescents (*d* = .25, *p* < .001), but no sex differences were found for externalizing problems (*d* = .06, *p* = .361).

The most prevalent outcome was no formal employment (53.4%), followed by IPV (any pattern, 43.3%), incomplete secondary schooling (33.6%), not being in a serious relationship during the last year (29.9%), welfare receipt (30.4%), and criminality (27.2%) (Table 2). With the exception of HIV, we found significant sex differences in the distribution of outcomes. Incomplete secondary school, no formal employment, criminality, substance use, social isolation, IPV (abuse and victimization and abuse only), and not being in a serious relationship were significantly more prevalent among male than female adolescents, whereas welfare receipt and psychological distress were significantly more prevalent among female adolescents.

**Table 2:** Distribution of adult outcomes in the whole sample and for men and women separately

Educational and employment	Whole sample (N = 1,391)	Men only (N = 661)	Women only (N = 730)	Men/Women comparison
	n (%)	n (%)	n (%)	RR (95% CI)
Incomplete secondary education	468 (33.6)	269 (40.7)	199 (27.3)	1.49 (1.28–1.73)
No formal employment	743 (53.4)	386 (58.4)	357 (48.9)	1.19 (1.08–1.32)
Welfare benefit	299 (29.3)	6 (1.3)	293 (52.7)	.02 (.01–.05)
Psychosocial				
Psychological distress	253 (18.2)	76 (11.5)	177 (24.2)	.47 (.37–.61)
Criminality	382 (27.5)	293 (44.3)	89 (12.2)	3.64 (2.94–4.50)
Substance use	367 (26.4)	268 (40.5)	99 (13.6)	2.99 (2.44–3.67)
Interpersonal				
Social isolation	150 (10.8)	92 (13.9)	58 (7.9)	1.75 (1.28–2.39)
No serious relationship	420 (30.2)	245 (37.1)	171 (23.4)	1.64 (1.39–1.93)
Intimate partner violence				
Victim and abuser	269 (27.1)	131 (30.9)	138 (24.3)	1.49 (1.11–1.99)
Victim	74 (7.5)	44 (10.4)	30 (5.3)	2.30 (1.40–3.77)
Abuser	86 (8.7)	30 (7.1)	56 (9.9)	.84 (.52–1.35)
HIV				
HIV positive	213 (15.3)	85 (12.9)	128 (17.5)	.73 (.57–.94)

CI = confidence interval.

The table reports the mean number of outcomes and the prevalence of each outcome in the whole sample and stratified by sex. Sex differences are expressed as risk ratios (RRs) for men compared with women. Estimated from one multiple-imputed data set to ensure coherence. All *p* values for men/women comparisons (chi-square) are < .001, except for intimate partner violence abusers (*p* = .590), and HIV (*p* < .016).

### Adolescent mental health problems and human capital outcomes

**Figure 2:**
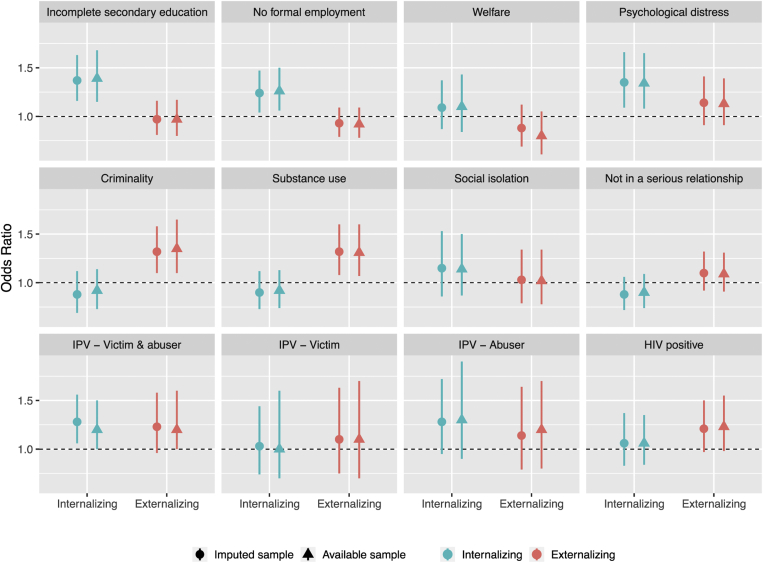
Adjusted associations between adolescent internalizing and externalizing problems and adult human capital outcomes. The figure shows in the y-axis, the adjusted odds ratio for the association of high psychological problems (vs. low) with adult outcomes for the whole sample and for men (blue) and women (red) separately. The central dot represents the estimated odds ratio, and the bar represents the 95% confidence intervals. The dotted line represents the null association. We reported fully adjusted estimates (see Supplementary Table 2 for the exact numbers). In addition, the figure shows associations for the imputed sample (round dot) and for the available sample (triangle) to show consistency of the estimates across these two methods for handling missing data. IPV = intimate partner violence.

Adolescents with high internalizing problems were more likely to experience adverse educational and employment outcomes in adulthood than those with low internalizing problems (Table 3 and Figure 2). ORs for incomplete secondary education and no formal employment at the age of 28 years were 1.37 (1.16–1.63) and 1.24 (1.04–1.47), respectively, for each standard deviation increase in the internalizing score. Adolescents reporting high internalizing problems were also more likely to report high psychological distress in adulthood compared with those with fewer problems (OR, 1.37; confidence interval [CI], 1.16–1.63). No association was found for welfare receipt. Adolescents with high externalizing problems were more likely to report criminality (OR, 1.32; CI, 1.10–1.58) and substance use (OR, 1.32; CI, 1.08–1.60) in adulthood but not psychological distress or poorer educational and employment outcomes after adjustment for internalizing problems and covariates (OR, 1.14; CI, .91–1.41). Adolescents with high internalizing problems were more likely than those with fewer problems to experience “abuse and victimization” and “abuse only” patterns of IPV in adulthood, but these differences only showed a trend toward significance in fully adjusted analyses (*p* = .065). The same result was found for the association between adolescent externalizing problems and “abuse and victimization” IPV in adulthood (*p* = .064 in adjusted analysis). No association was found between adolescent externalizing or internalizing problems and adult social isolation, serious relationship, and HIV status.

**Table 3:** Association between adolescent internalizing and externalizing problems with human capital outcomes

	Internalizing				Externalizing				Nagelkerke R^2^
	Sex-adjusted	*P*	Fully adjusted	*P*	Sex-adjusted	*P*	Fully adjusted	*P*	Fully adjusted
Education andemployment									
Incomplete secondary education	1.38 (1.21–1.59)	.001	1.37 (1.16–1.63)	.004	1.07 (.93–1.23)	.440	.97 (.81–1.16)	.756	.19
No formal employment	1.21 (1.05–1.39)	.005	1.24 (1.04–1.47)	.010	.99 (.87–1.13)	.919	.93 (.79–1.09)	.373	.09
Welfare benefit	1.06 (.89–1.26)	.460	1.09 (.87–1.37)	.423	.89 (.73–1.08)	.260	.88 (.69–1.12)	.311	
Psychosocial									
Psychological distress	1.37 (1.16–1.63)	.001	1.35 (1.09–1.66)	.008	1.25 (1.04–1.49)	.012	1.14 (.91–1.41)	.254	.52
Criminality	1.02 (.85–1.23)	.653	.88 (.69–1.12)	.284	1.23 (1.06–1.43)	.011	1.32 (1.10–1.58)	.012	.14
Substance use	1.03 (.88–1.21)	.640	.90 (.73–1.12)	.372	1.27 (1.09–1.49)	.002	1.32 (1.08–1.60)	.004	.21
Interpersonal									
Social isolation	1.17 (.93–1.48)	.128	1.15 (.86–1.53)	.233	1.07 (.86–1.33)	.518	1.03 (.79–1.34)	.871	.17
Not in a serious relationship	.94 (.81–1.09)	.410	.88 (.72–1.06)	.161	1.03 (.89–1.19)	.760	1.10 (.92–1.32)	.313	.06
Intimate partner violence									
Victim and abuser	1.40 (1.15–1.70)	.003	1.24 (.96–1.59)	.065	1.28 (1.06–1.56)	.047	1.23 (.96–1.58)	.064	.13
Victim	1.01 (.72–1.43)	.939	.97 (.65–1.44)	.940	1.03 (.74–1.44)	.974	1.10 (.75–1.63)	.707	
Abuser	1.44 (1.08–1.93)	.019	1.33 (.95–1.86)	.072	1.28 (.95–1.72)	.201	1.14 (.79–1.64)	.449	
HIV									
HIV positive	1.20 (.99–1.45)	.054	1.06 (.83–1.37)	.481	1.16 (.98–1.38)	.098	1.21 (.97–1.50)	.106	.10

The table shows the fold-increase in the odds (OR, odds ratio) of each adult human capital outcomes (rows) for each SD increase in adolescent internalizing and externalizing problems (columns). In fully adjusted model, internalizing and externalizing symptoms are entered in the same model together with the selected covariates.

### Complementary analyses

Analyses for the imputed and nonimputed samples yielded virtually identical results (Figure 2 and Appendix Tables 2 and 3). Stratified analyses by sex showed only minor sex differences (Supplementary Table S2 and S3).

## Discussion

To our knowledge, this is the first study to investigate associations between adolescent mental health problems and adult human capital outcomes in a population-based study in sub-Saharan Africa across an extended time scale. Using data from a large 28-year South African birth cohort, we found that adolescents with internalizing and externalizing problems were likely to experience a range of negative adult outcomes, with associations being in the same directions for both male and female adolescents. Externalizing problems were associated with substance use and criminality and internalizing problems with incomplete schooling and lack of formal employment.

### Adolescent mental health problems and educational and employment outcomes

Internalizing problems in adolescence were linked to adverse educational and employment outcomes in young adulthood, consistent with previous studies reporting small but significant effects of internalizing problems on education and employment [26,27]. However, no association was found for externalizing problems. This contrasts with longitudinal research from HICs reporting that externalizing problems are more consistently associated with lower educational outcomes than internalizing problems [28,29]. These differences may relate to South Africa's sociopolitical context characterized by generalized poverty, high levels of violence, and a fragmented and underperforming education system. Approximately 60% of youths leave secondary school without graduating [30], with very high rates of youth unemployment [31]. Rather, we found that individual differences in education and employment are explained by internalizing problems, affecting men more than women. Internalizing problems, such as anxiety and depression, are typically associated with withdrawal from the outside world, hopelessness, and learned helplessness [18]. These mental states may reduce psychological resources necessary to proactively engage with challenging socioeconomic conditions and find, or take advantage of, education and job opportunities.

### Adolescent mental health problems and psychosocial outcomes

High adolescent internalizing problems were associated with higher levels of psychological distress characterized by depression, anxiety, and somatic problems, whereas high externalizing problems were associated with criminality and substance use. This finding, in line with reports from other countries [28,[32], [33], [34]], delineates a pattern of homotypic continuity suggesting that adolescent internalizing and externalizing problems evolve into more severe mental health and social outcomes later in life. Studies have found that both causation and social selection processes are at play in explaining such associations [35]. Improved understanding of mechanisms leading to continuity of problems in LMIC contexts would be helpful to inform preventive actions. In particular, homotypic continuity suggests that future studies in the same context should aim to clarify the role of the external environment and social responses to internalizing and externalizing problems. The inner-directed nature of internalizing problems is more difficult to detect and may remain unrecognized and unaddressed, resulting in persistent and cumulative consequences. In turn, noncompliance and aggression associated with externalizing problems may elicit punitive reactions from parents, peers, and teachers, which could exacerbate problems in social relationships and adjustment.

### Adolescent mental health problems and interpersonal outcomes

IPV, as a victim and/or an abuser, was reported by close to half of all participants, with the most frequent pattern among men and women being both perpetrator and victim. Interpersonal violence is a national problem in South Africa. It is the second leading cause of death and disability-adjusted life years, with 184 per 100,000 homicide deaths among young men aged 15–29 years [36]. There are also exceedingly high rates of sexual coercion and rape of women [37]. Rates of IPV are high among both men and women and, similar to our findings, men and women report roughly equal rates of perpetration and of victimization [38,39]. In prior South African studies, witnessing parental violence and experiencing severe physical punishment have been identified as predictors of IPV, together with substance abuse and low socioeconomic status [38,40].

Our finding of a lack of association between internalizing and externalizing problems and partnership status in young adulthood has been noted elsewhere [28], though some studies have demonstrated stronger effects of adolescent mental health problems on the quality of adult romantic relationships when subsets of problems are analyzed, such as depressive symptoms [41]. Social isolation and absence of a serious relationship in the past 12 months were significantly higher among men than among women. Cultural customs in South Africa, such as the payment of a bride price [42], as well as poverty and unemployment inhibit cohabitation and delay marriage [43]. Young women tend to remain in their parental home, whereas men rent rooms alone or with others. Many have migrated to urban areas in search of work, leaving families behind in rural areas [44]. This provides some explanation for a heightened sense of social isolation driven by economic, structural, and cultural factors.

We found very high rates of adverse outcomes in this cohort, the most common being unemployment, IPV, incomplete secondary education, absence of a serious relationship, welfare receipt, and criminality ‒ together affecting at least one in four individuals. Men, at 28 years of age, were more likely to report incomplete secondary education, no formal employment, criminality and substance use, social isolation, and not being in a serious relationship. Women were more likely to be receiving social welfare and experiencing psychological distress. These sex differences are consistent with prior research documenting growing advantages for girls and women in education, increasing impacts on employment [45], higher rates of psychological distress among women than among men [46], and generally higher rates of criminality and substance use among men [47]. Welfare receipt in this context refers to a government cash transfer available to primary caregivers of children who qualify through an income means test. While both parents are eligible to receive welfare, the greatest number of recipients is women [48]. Finally, our finding of no sex difference in HIV is consistent with South African data indicating that infections start earlier among young women but become similar across sexes by the age of 30 years [49].

### Strengths and limitations

This study used data from the longest running birth cohort in sub-Saharan Africa, assessed a wide range of outcomes encompassing most human capital domains, and examined longitudinal associations between adolescent mental health and human capital. It focuses on the high prevalence of poor adult outcomes, many of which are associated with mental health problems evident during adolescence. However, it is important to acknowledge the following limitations. First, as in all birth cohort studies [7], there is sample attrition arising from a combination of factors, including local migration patterns, described in detail elsewhere [17]. Second, adolescent mental health problems and human capital outcomes were both self-reported, which is known to inflate associations owing to shared method variance. A related limitation is the possibility of social desirability bias found in all self-report measures, especially in reporting criminal or violent behaviors and potential gendered responses. Finally, given the complex nature of the development of internalizing and externalizing problems and the many factors – including genetic – that influence pathways, some of which not included in this study, the ORs in our models should be interpreted as measures of association and do not necessarily imply causation.

## Conclusion

Despite substantial research conducted to understand associations between adolescent mental health problems and later adult human capital outcomes in HICs, little attention is given to the personal and societal consequences of adolescent mental health problems for adult outcomes in low- to middle-income, non-Western settings. This study, the first in sub-Saharan Africa, shows that adverse outcomes are highly prevalent, more common among men, and associated with adult mental health problems detectable in adolescence among both men and women. Consonant with literature from HICs, this study confirms that mental health problems in adolescence are linked to a range of adverse human capital outcomes in the long term. Our study contributes to extant literature by showing that these associations hold also in conditions of substantial adversity and socioeconomic disadvantage. Taken together, our findings call for early prevention and intervention targeting adolescent mental health problems in LMICs. The specificity of outcomes for internalizing versus externalizing problems highlights the need for careful assessment of the nature of adolescence mental health problems, particularly less visible but equally harmful internalizing problems, to provide contextually appropriate interventions coupled with efforts to address social, cultural, and economic factors.

In South Africa, most resources are directed at structural issues associated with LMICs, primarily unemployment, poverty, and skill deficits in adulthood, when human capital outcomes have already been cemented for a large proportion of the population. More focus needs to be given to addressing adolescent mental health before these problems lead to more fixed consequences.

## Funding Sources

The adult outcome data collection at 28 years of age was funded by the 10.13039/100000865Bill & Melinda Gates Foundation, Seattle, WA (OPP1164115). The funder played no role in the preparation of the manuscript, analysis of the data, interpretation and discussion of the results, or the decision to submit the manuscript for publication.
