# Supplementary material for: Adolescent Mental Health Problems and Adult Human Capital: Findings From the South African Birth to Twenty Plus Cohort at 28 Years of Age
Source: J Adolesc Health. 2021 Nov;69(5):782–9. doi: 10.1016/j.jadohealth.2021.04.017 (PMC8552796; doi:10.1016/j.jadohealth.2021.04.017)
Supplement: Supplementary Tables [file mmc1.pdf]

Appendix

Table A. Missing data on the outcome variables

Outcome variable	N. valid observations	N. missing observations	% missing observations
Psychological problems	1391	0	0
Social isolation	1391	0	0
Incomplete secondary education	1381	10	0.7
Criminality	1357	34	2.4
No serious relationship	1353	38	2.7
Substance use	1327	64	4.6
No formal employment	1257	134	9.6
HIV positive	1256	135	9.7
Welfare	1021	370	26.6
Intimate partner violence	991	400	28.8

Table B. Association between adolescent internalizing and externalizing problems with human capital outcomes by sex in the imputed sample (N=1391)

	Males				Females			
	Internalizing		Externalizing		Internalizing		Externalizing	
	Sex-adjusted	Fully adjusted	Sex-adjusted	Fully adjusted	Sex-adjusted	Fully adjusted	Sex-adjusted	Fully adjusted
**Education & employment**								
Incomplete secondary education	1.42 (1.17-1.72)	1.45 (1.14-1.85)	1.04 (0.87-1.25)	0.91 (0.72-1.16)	1.35 (1.12-1.64)	1.34 (1.05-1.7)	1.1 (0.88-1.36)	1.04 (0.79-1.37)
No formal employment	1.31 (1.06-1.63)	1.26 (0.98-1.64)	1.09 (0.9-1.32)	1.02 (0.81-1.27)	1.13 (0.96-1.34)	1.22 (0.99-1.51)	0.91 (0.76-1.08)	0.85 (0.69-1.06)
Welfare	1.02 (0.36-2.85)	1.00 (0.18-5.58)	0.98 (0.37-2.63)	0.83 (0.16-4.34)	1.06 (0.88-1.27)	1.1 (0.87-1.39)	0.88 (0.72-1.08)	0.87 (0.68-1.13)
**Psychosocial**								
Psychological distress	1.42 (1.06-1.9)	1.37 (0.95-1.97)	1.27 (0.96-1.69)	1.18 (0.83-1.67)	1.35 (1.11-1.66)	1.34 (1.05-1.72)	1.23 (1.00-1.52)	1.12 (0.87-1.45)
Criminality	1.03 (0.82-1.29)	0.87 (0.66-1.16)	1.25 (1.04-1.5)	1.32 (1.06-1.66)	1.01 (0.77-1.33)	0.9 (0.63-1.28)	1.2 (0.93-1.54)	1.29 (0.93-1.79)
Substance use	1.03 (0.83-1.27)	0.89 (0.67-1.18)	1.3 (1.07-1.59)	1.35 (1.05-1.74)	1.04 (0.81-1.33)	0.92 (0.67-1.26)	1.23 (0.97-1.56)	1.29 (0.96-1.72)
**Interpersonal**								
Social isolation	1.09 (0.79-1.5)	1.13 (0.76-1.68)	0.95 (0.71-1.28)	0.91 (0.64-1.3)	1.28 (0.96-1.72)	1.17 (0.82-1.69)	1.25 (0.93-1.69)	1.21 (0.84-1.74)
Not in a serious relationship	0.97 (0.79-1.2)	0.91 (0.7-1.19)	1.01 (0.83-1.23)	1.05 (0.83-1.34)	0.91 (0.74-1.11)	0.84 (0.65-1.07)	1.05 (0.85-1.29)	1.15 (0.89-1.47)
Intimate Partner Violence								
Victim & Abuser	1.32 (1.00-1.7)	1.10 (0.80-1.60)	1.32 (1.00-1.70)	1.31 (0.90-1.90)	1.44 (1.10-1.80)	1.35 (1.00-1.80)	1.25 (1.00-1.60)	1.21 (0.90-1.70)
Victim	1.05 (0.70-1.60)	1.00 (0.60-1.80)	1.04 (0.70-1.60)	1.08 (0.60-1.90)	0.98 (0.60-1.50)	0.87 (0.50-1.60)	1.06 (0.70-1.60)	1.22 (0.70-2.20)
Abuser	1.30 (0.80-2.20)	1.09 (0.60-2.00)	1.34 (0.80-2.20)	1.26 (0.70-2.30)	1.54 (1.10-2.10)	1.42 (1.00-2.10)	1.29 (0.90-1.80)	1.12 (0.70-1.70)
**HIV**								
HIV positive	1.31 (0.97-1.76)	1.21 (0.82-1.80)	1.14 (0.87-1.48)	1.08 (0.77-1.51)	1.14 (0.91-1.42)	0.99 (0.74-1.33)	1.18 (0.94-1.47)	1.31 (0.98-1.76)

Associations are expressed as odds ratios

Table C. Association between adolescent internalizing and externalizing problems with human capital outcomes by sex in the available sample (N=896)

	Males				Females			
	**Internalizing**		**Externalizing**		**Internalizing**		**Externalizing**	
	Sex-adjusted	Fully adjusted	Sex-adjusted	Fully adjusted	Sex-adjusted	Fully adjusted	Sex-adjusted	Fully adjusted
**Education & employment**								
Incomplete secondary education	1.46 (1.16-1.83)	1.54 (1.15-2.07)	1.01 (0.83-1.24)	0.88 (0.67-1.15)	1.32 (1.09-1.61)	1.3 (0.99-1.69)	1.12 (0.91-1.37)	1.1 (0.83-1.44)
No formal employment	1.39 (1.10-1.75)	1.34 (1.00-1.78)	1.12 (0.92-1.38)	1.05 (0.81-1.35)	1.1 (0.92-1.31)	1.2 (0.96-1.50)	0.86 (0.71-1.04)	0.83 (0.66-1.04)
Welfare	1.18 (0.37-3.79)	NA	0.79 (0.23-2.76)	NA	1.02 (0.83-1.25)	1.1 (0.84-1.44)	0.81 (0.65-100)	0.8 (0.61-1.06)
**Psychosocial**								
Psychological distress	1.46 (1.07-2)	1.43 (0.94-2.18)	1.27 (0.95-1.69)	1.21 (0.82-1.78)	1.32 (1.08-1.61)	1.3 (1.01-1.68)	1.18 (0.96-1.45)	1.11 (0.85-1.44)
Criminality	1.11 (0.89-1.38)	0.91 (0.69-1.21)	1.31 (1.07-1.61)	1.34 (1.03-1.72)	1.04 (0.78-1.41)	0.92 (0.63-1.35)	1.24 (0.94-1.65)	1.36 (0.95-1.96)
Substance use	1.09 (0.87-1.35)	0.89 (0.67-1.18)	1.38 (1.12-1.71)	1.4 (1.08-1.82)	1.04 (0.79-1.36)	0.95 (0.66-1.35)	1.19 (0.91-1.55)	1.2 (0.86-1.68)
**Interpersonal**								
Social isolation	1.02 (0.74-1.40)	1.14 (0.76-1.71)	0.85 (0.62-1.16)	0.79 (0.52-1.18)	1.34 (0.99-1.81)	1.14 (0.77-1.68)	1.34 (0.99-1.82)	1.33 (0.9-1.95)
Not in a serious relationship	0.96 (0.77-1.21)	0.92 (0.69-1.23)	0.97 (0.79-1.20)	1.01 (0.78-1.32)	0.94 (0.75-1.16)	0.87 (0.66-1.14)	1.09 (0.88-1.36)	1.17 (0.9-1.52)
Intimate Partner Violence								
Victim & Abuser	1.30 (0.90-1.80)	0.99 (0.60-1.50)	1.3 (1.00-1.80)	1.43 (1.00-2.10)	1.43 (1.10-1.80)	1.39 (1.00-1.90)	1.23 (1.00-1.6)	1.22 (0.90-1.70)
Victim	1.10 (0.70-1.80)	1.06 (0.50-2.10)	1.10 (0.70-1.70)	1.08 (0.6-2.00)	1.00 (0.60-1.70)	0.90 (0.50-1.70)	1.03 (0.60-1.70)	1.18 (0.60-2.20)
Abuser	1.20 (0.60-2.20)	0.89 (0.40-2.20)	1.30 (0.80-2.30)	1.39 (0.60-3.10)	1.49 (1.00-2.10)	1.45 (0.90-2.30)	1.23 (0.90-1.80)	1.06 (0.70-1.70)
**HIV**								
HIV positive	1.39 (1.02-1.90)	1.29 (0.86-1.93)	1.15 (0.86-1.53)	1.09 (0.74-1.59)	1.10 (0.88-1.38)	0.97 (0.71-1.32)	1.17 (0.93-1.47)	1.36 (1.00-1.87)

Associations are expressed as odds ratios; NA, not available
